# Broadband achromatic dielectric metalenses

**DOI:** 10.1038/s41377-018-0078-x

**Published:** 2018-11-07

**Authors:** Sajan Shrestha, Adam C. Overvig, Ming Lu, Aaron Stein, Nanfang Yu

**Affiliations:** 1 Department of Applied Physics and Applied Mathematics, Columbia University, New York, NY 10027 USA; 2 Brookhaven National Laboratory, Center for Functional Nanomaterials, Upton, NY 11973 USA

## Abstract

Metasurfaces offer a unique platform to precisely control optical wavefronts and enable the realization of flat lenses, or metalenses, which have the potential to substantially reduce the size and complexity of imaging systems and to realize new imaging modalities. However, it is a major challenge to create achromatic metalenses that produce a single focal length over a broad wavelength range because of the difficulty in simultaneously engineering phase profiles at distinct wavelengths on a single metasurface. For practical applications, there is a further challenge to create broadband achromatic metalenses that work in the transmission mode for incident light waves with any arbitrary polarization state. We developed a design methodology and created libraries of meta-units—building blocks of metasurfaces—with complex cross-sectional geometries to provide diverse phase dispersions (phase as a function of wavelength), which is crucial for creating broadband achromatic metalenses. We elucidated the fundamental limitations of achromatic metalens performance by deriving mathematical equations that govern the tradeoffs between phase dispersion and achievable lens parameters, including the lens diameter, numerical aperture (NA), and bandwidth of achromatic operation. We experimentally demonstrated several dielectric achromatic metalenses reaching the fundamental limitations. These metalenses work in the transmission mode with polarization-independent focusing efficiencies up to 50% and continuously provide a near-constant focal length over *λ* = 1200–1650 nm. These unprecedented properties represent a major advance compared to the state of the art and a major step toward practical implementations of metalenses.

## Metalenses: sharpening the focus of new technology

Small, high-performance imaging systems could be built using flat lenses made from specially arranged nanoscale pillars. Traditional lenses rely on the curvature and thickness of glass to focus light, but metalenses, which can be smaller, thinner, and more flexible, have surfaces comprised of thousands of nanoscale pillars whose geometries are carefully designed to control optical phase. However, problems still arise in maintaining the same focal length across a wide wavelength range, leading to image blurring. Now, Nanfang Yu at Columbia University in New York, USA, and co-workers have designed a library of meta-units—the nano-pillars used to create metalenses—with several different cross-sectional geometries. They have combined these meta-units in various patterns to build broadband metalenses, which exhibit consistent focal length across a broad near-infrared wavelength range, significantly improving the final image quality. Furthermore, such metalenses work in the transmission mode and can focus light of any arbitrary polarization state.

## Introduction

Expanding the control of optical wavefronts is the promise of a class of engineered two-dimensional materials called “metasurfaces”^1–6^. Composed of subwavelength scatterers with tailored optical responses, or “meta-units”, a metasurface can realize a variety of device functions^7–26^ with a completely flat form-factor if the library of meta-units is sufficiently diverse. The flat form-factor is a particular advantage for devices such as lenses, enabling compact imaging systems to be fabricated with complementary metal oxide semiconductor (CMOS)-compatible processes.

However, as with lenses based on bulk materials, metasurface lenses^27–39^, or “metalenses”, must be designed to minimize aberrations for use in high-performance imaging systems. Recent work^40,41^ has substantially reduced monochromatic aberrations by using two parallel metasurfaces, yielding compound metalenses with a large field of view. Initial efforts to correct chromatic aberrations inherent to diffractive optical systems have led to demonstrations of multiwavelength metalenses^42–45^. More recent endeavors have focused on extending the correction to a continuous range of wavelengths but have been limited to reflective lenses^46–49^, polarization-dependent focusing^48–50^, and/or limited operation bandwidths^46,47^.

Here we introduce a CMOS-compatible platform achieving diffraction-limited, polarization-independent focusing in the transmission mode across a broad bandwidth (up to Δ*λ* = 450 nm) in the near-infrared wavelength range. We clarify the role of the spectral degree of freedom^42,51^, *C*(*ω*), which determines the reference phase at each frequency. We incorporate this understanding into a framework for dispersion-engineered metalenses that maps the design challenge onto filling a parameter space we call “phase-dispersion” space. We introduce novel meta-unit geometries that fill this space to a much greater degree than conventional meta-unit geometries. Using this new framework, we explore the fundamental limitations of chromatic aberration correction in metalenses and experimentally implement several metalenses reaching these limits.

Chromatic dispersion is the dependence of focal length on the wavelength of light. Conventional, bulky lenses are based on refraction and exhibit positive dispersion (higher frequencies have smaller focal lengths). Diffractive focusing elements (such as Fresnel zone plates) exhibit the opposite (negative) dispersion^52^. In imaging systems, both types of dispersion lead to a degradation of image quality due to blurring, an effect known as chromatic aberration. In both schemes, chromatic aberration correction can be achieved by careful design of a composite system of numerous optical elements, with the drawbacks of increased complexity, size, weight, and cost.

Diffractive lenses have an advantage over refractive lenses in that they are flat and lightweight and can be fabricated with conventional nanofabrication techniques at low cost. However, these lenses have a much lower focusing efficiency due to the presence of high diffractive orders. Metalenses can be thought of as diffractive lenses with only one diffractive order, which eliminates this disadvantage while inheriting all the advantages of a conventional diffractive element over their bulky, refractive counterparts. Furthermore, the meta-units comprising metalenses are vastly more tailorable than those that make up simple diffractive elements (e.g., gratings), opening up the possibility to correct chromatic aberration in a single optical element.

## Results

In its most general form, a broadband achromatic metasurface presents a daunting challenge. Each meta-unit must be designed to simultaneously satisfy the phase requirement at all design wavelengths. Since the phase profiles for each frequency are potentially entirely independent, each meta-unit should provide a unique phase response (never to be re-used elsewhere in the metasurface). The size of the meta-unit library will therefore generally be equal to the number of elements of the metasurface. This fact compels the careful, joint consideration of meta-unit library design combined with the optical functionality desired.

**Fig. 1:**
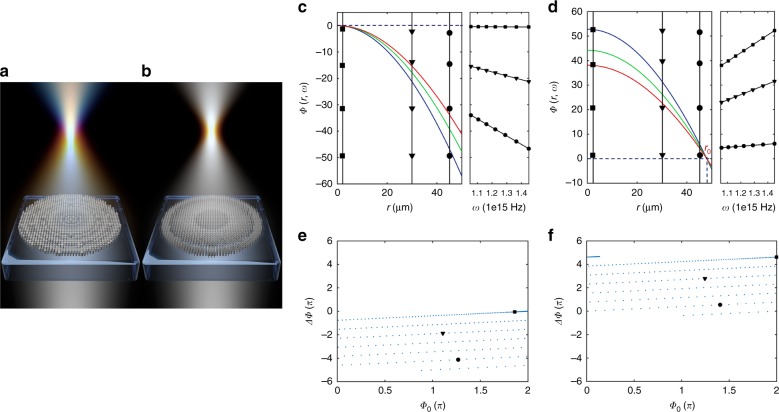
Comparison between monochromatic metasurface lenses and achromatic lenses. **a** Schematic of a monochromatic metalens composed of simple cylindrical meta-units, showing diffractive dispersion (focal length proportional to frequency). **b** Schematic of a broadband achromatic metalens composed of meta-units with complex cross sections, showing dispersionless focusing. **c** Spatial (left panel) and spectral (right panel) phase profiles required for a sample achromatic metalens (radius of 50 µm, focal length of 100 µm, operating in the wavelength range of *λ* = 1.3–1.8 µm) designed with the conventional choice of $C\left( \omega \right) = \frac{\omega }{c}f$. Three different frequencies are represented by three colors, and three positions are represented by different symbols. **d** Similar diagrams as in **c** but for our choice of $C\left( \omega \right) = \frac{\omega }{c}\sqrt {r_0^2 + f^2}$. **e**, **f** Requirements of meta-units for the metalens in the phase-dispersion space, where *ϕ*_0_ is the phase of the smallest frequency and the dispersion ${\mathrm{\Delta }}\phi = \frac{{{\mathrm{d}}\phi }}{{{\mathrm{d}}\omega }}{\mathrm{\Delta }}\omega$ for a given bandwidth Δ*ω* is the difference in phase between the largest and smallest frequencies

For converging achromatic metalenses, the spatial and spectral phase profiles follow a simple relation up to a spectral degree of freedom, *C*(*ω*):

1 $$\phi \left( {r,\omega } \right) = - \frac{\omega }{c}\left( {\sqrt {r^2 + f^2} } \right) + C\left( \omega \right)$$

where *f* is the focal length, *r* is the radial position, *c* is the speed of light, and *ω* is the angular frequency. The conventional choice is $C\left( \omega \right) = \frac{\omega }{c}f$, which renders the required phase 0 for all frequencies at the center of the lens (*r* = 0), a convenient and intuitive option. Figure 1 summarizes the difference between the conventional design approach, which achieves focusing with chromatic aberration (Fig. 1a), and a novel design approach introduced herein, which produces dispersionless focusing (Fig. 1b). Figure 1c depicts the required spatial phase profiles based on the conventional choice for three select frequencies, as well as the spectral phase profiles (phase dispersion) at three selected positions along the metalens. We propose a generalized choice of

2 $$C\left( \omega \right) = \frac{\omega }{c}\sqrt {r_0^2 + f^2} + C_0$$

which makes the required phase $\phi \left( {r_0,\omega } \right) = C_0$ for all frequencies at the reference position *r* = *r*_0_. Figure 1d depicts the equivalent phase profiles of Fig. 1c for comparison.

To understand the impact of this choice, we first note that Eq. 1 is a simple linear function with respect to frequency. This feature suggests parameterizing the spectral phase required at each position as

3 $$\phi \left( {r,\omega } \right) = \phi _0\left( r \right) + \frac{{{\mathrm{d}}\phi }}{{{\mathrm{d}}\omega }}\left( r \right)\left( {\omega - \omega _0} \right)$$

where *ϕ*_0_(*r*) is the phase at a reference frequency *ω*_0_ and $\frac{{{\mathrm{d}}\phi }}{{{\mathrm{d}}\omega }}(r)$ is the dispersion required at that frequency. In other words, a given meta-unit to be placed at position *r* can be described by two parameters: phase, *ϕ*_0_, and dispersion, $\frac{{{\mathrm{d}}\phi }}{{{\mathrm{d}}\omega }}$. This aspect motivates the exploration of both meta-unit libraries and the choice of *C*(*ω*) in a parameter space defined by these two quantities, which we call “phase-dispersion” space. At each position on a metalens, Eq. 1 specifies the required values of phase and dispersion relative to a reference phase and a reference dispersion set by *C*(*ω*). These requirements can be plotted in the phase-dispersion space to visualize the extent of parameter space that a meta-unit library must fill to create the metalens (Fig. 1e, f).

**Fig. 2:**
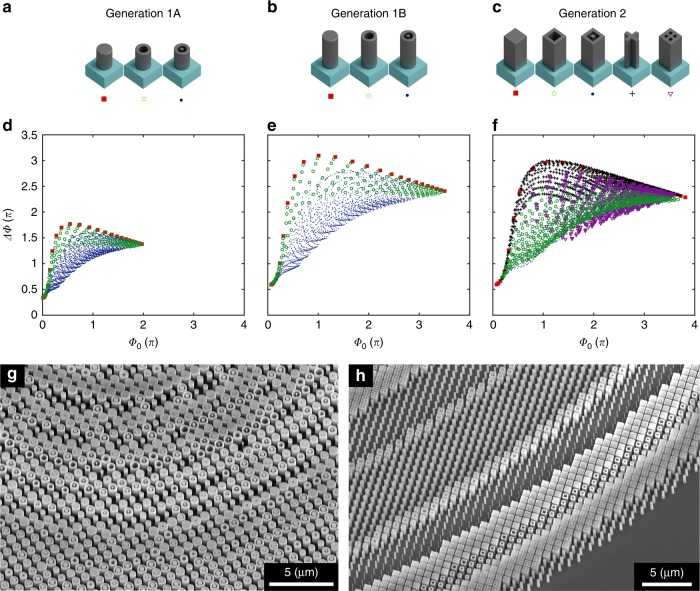
Meta-unit libraries. **a**–**c** Schematics showing meta-unit archetypes, each representing a subclass of meta-units composed of the archetype’s basic shape but with varying in-plane geometrical parameters (e.g., inner and outer radii of annular pillars). **d** Calculated phase, Φ_0_, of the lowest frequency (or largest wavelength, *λ* = 1.6 µm) and dispersion, ${\mathrm{\Delta \Phi }} = \frac{{{\mathrm{d}}\phi }}{{{\mathrm{d\omega }}}}{\mathrm{\Delta }}\omega$, for the chosen bandwidth Δ*ω* (i.e., *λ* = 1.2–1.6 µm) of each meta-unit used in the Generation 1A library: singular pillars (red squares), annular pillars (green circles), and concentric rings (blue dots). The meta-units are composed of silicon, with a height of 800 nm. Note the expansive coverage of phase-dispersion space compared to that obtained by employing only the conventional choice of singular pillars. **e** Calculated phase and dispersion for the Generation 1B library, differing from the Generation 1A library only by increasing the height to 1400 nm. The range of dispersion achieved is nearly doubled. **f** Calculated phase and dispersion for the Generation 2 library, keeping the same height as the Generation 1B library but switching to archetypes with four-fold symmetry instead of rotational symmetry. This change expands the number of archetypes to include crosses (black crosses) and inscribed crosses (purple triangles). The two orange curves define the upper and lower boundaries that can be reached by dielectric meta-units, found by an exhaustive computational search (see Supplementary Information Section VIII). Our choice of meta-unit cross sections fully populates the possible area in the phase-dispersion space, while being feasible for nanofabrication. **g**, **h** Scanning electron microscope images of fabricated metalenses using Generation 1A and Generation 2 meta-units, respectively

Figure 2 complements Fig. 1 by summarizing the area in the phase-dispersion space achievable by our meta-unit libraries. Figure 2a–c shows three meta-unit libraries based on dielectric pillars of a variety of cross-sectional shapes, and Fig. 2d–f maps the coverage of these libraries in the phase-dispersion space. Note in particular that all values of dispersion are positive, rendering the conventional choice of $C\left( \omega \right) = \frac{\omega }{c}f$ incompatible, as it prescribes only negative values of dispersion relative to the center of the metalens (Fig. 1e). In contrast, the proposed form of *C*(*ω*) prescribes positive values of dispersion by setting the reference as a position *r*_0_ (Fig. 1f and Supplementary Information Sections I and II). The dielectric libraries can now match the required phase over a continuous bandwidth for positions *r* < *r*_0_ but not outside of that domain. This suggests making *r*_0_ the radius of the metalens, which fixes the dispersion required at the edge of the metalens $\left. {\frac{{{\mathrm{d}}\phi }}{{{\mathrm{d}}\omega }}} \right|_{\min }$ as zero and causes the dispersion to increase toward the center of the lens. Consequently, the dispersion required at the center of the lens $\left. {\frac{{{\mathrm{d}}\phi }}{{{\mathrm{d}}\omega }}} \right|_{\max }$ monotonically increases with metalens radius.

The range of phase dispersion, ${\mathrm{\Delta \Phi }}^\prime = \left( {\left. {\frac{{{\mathrm{d}}\phi }}{{{\mathrm{d}}\omega }}} \right|_{{\mathrm{max}}} - \left. {\frac{{{\mathrm{d}}\phi }}{{{\mathrm{d}}\omega }}} \right|_{{\mathrm{min}}}} \right){\mathrm{\Delta }}\omega$, covered by a meta-unit library therefore limits the maximum radius *R*_max_ of an achromatic metalens that can be achieved with that library. Specifically, this limitation is given by the following relation (details in Supplementary Information Section III):

4 $$R_{{\mathrm{max}}} \le \frac{{\Delta \Phi ^\prime {{c}}}}{{\Delta \omega \left( {\frac{1}{{{\mathrm{NA}}}} - \sqrt {\frac{1}{{{\mathrm{NA}}^2}} - 1} } \right)}}$$

which simplifies to

5 $$R_{{\mathrm{max}}}{\mathrm{NA}}{\mathrm{\Delta }}\omega \le 2{{c\Delta \Phi }}^\prime$$

when the numerical aperture NA « 1.

Equation 5 imposes the tradeoffs in this design problem and indicates that metalenses with a larger diameter, higher NA, and/or broader operational bandwidth, Δ*ω*, require a larger ΔΦ′ and therefore a more diverse meta-unit library. The challenge, then, is to populate our library with meta-units with the largest range of dispersive responses possible within a fabrication scheme.

To meet this challenge, we employ a dielectric metasurface platform of amorphous silicon nanostructures on a quartz substrate for proof-of-principle demonstrations. We model meta-units operating in transmission mode as dielectric waveguides with frequency-dependent effective refractive indices, $n_{{\mathrm{eff}}}\left( \omega \right)$ (Supplementary Information Section IV). For each frequency, $n_{{\mathrm{eff}}}\left( \omega \right)$ is calculated by eigenmode analysis on a finite difference grid and recorded for each choice of meta-unit cross-sectional shape. The phase is then obtained by an analytical thin film interference model with the meta-unit layer having an index of $n_{{\mathrm{eff}}}\left( \omega \right)$ and thickness of *H*. This technique differs from previous approaches^46,47^ that rely on sharp resonances exhibited by infinitely periodic meta-units (Supplementary Information Section V).

Figure 2a shows the first generation of our meta-unit library, called Generation 1A, with a thickness of *H* = 800 nm. Three archetypical cross sections are used: singular pillars, annular pillars, and concentric pillars. Each archetype is parameterized by the relevant radii and therefore represents a subclass of meta-units. It is evident that each subclass characteristically fills a different area of the phase-dispersion space (Fig. 2d). In particular, singular pillars (the conventional choice for dielectric meta-units) provide the highest dispersion for each phase value. However, this approach is limited to a singular value of dispersion for each phase, suggesting that the conventional meta-unit library composed of only this subclass is a poor choice. Annular pillars allow for varied and slightly lower values of dispersion for each phase in comparison, while concentric pillars allow for even lower values of dispersion (Fig. 2d). Together, the meta-unit library fills an appreciable area of the phase-dispersion space.

The extended coverage offered by these new archetypes can be understood as structural dispersion engineering^53^. High-dispersion meta-units (such as singular pillars) retain more short-wavelength light within the silicon compared to longer-wavelength light; in contrast, low-dispersion meta-units (such as concentric pillars) retain nearly the same amount of short- and long-wavelength light within the silicon, acting similar to dispersionless effective media (mode profiles of sample meta-units are provided in Supplementary Information Section IV).

Further improvement of the coverage of the phase-dispersion space, in particular, the extent of ΔΦ′, requires an increase in the height of the meta-units. Figure 2b shows meta-unit library Generation 1B. Its cross-section archetypes are identical to those of Generation 1A, but they differ in height, *H* = 1400 nm. A comparison of Fig. 2d, e reveals that taller meta-units greatly expand the coverage of the phase-dispersion space, at the cost of increased fabrication challenge.

Figure 2c depicts meta-unit library Generation 2. This library has the same height as Generation 1B but has four-fold symmetry rather than rotational symmetry. This reduction of symmetry allows for additional subclasses not available in rotationally symmetric schemes without sacrificing polarization-independent performance. In particular, we explore crosses and inscribed crosses as two additional archetypes. The former more densely populates the medium-high dispersion range, while the latter extends the lowest dispersion range at large phases (acting more like an effective material than any other archetype in that phase range) (Fig. 2f).

**Fig. 3:**
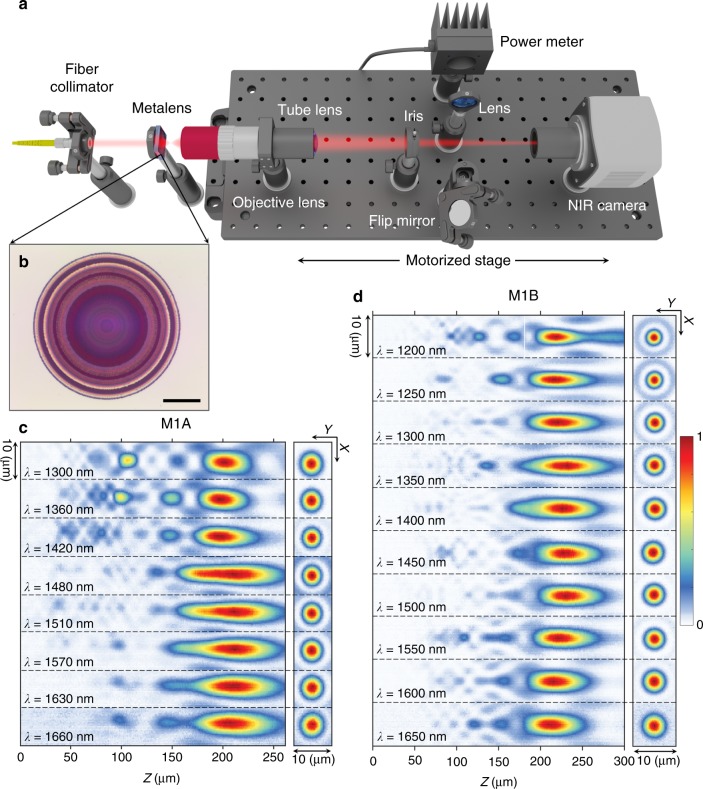
Experimental characterization of achromatic metalenses. **a** Schematic depicting the optical setup. The optics for mapping the 3D far-field of the metalenses are mounted on a motorized stage. A flip mirror allows light to pass from a target focal plane to a power meter for efficiency measurements. **b** Optical image of a sample metalens. Scale bar: 25 μm. **c** Measured far-field intensity distributions of metalens M1A, composed of Generation 1A meta-units and with diameter *D* = 100 μm and target focal length *f* = 200 μm, corresponding to NA = 0.24. Measured normalized intensity distributions in the axial plane (*x*–*z* cross section) are shown for select wavelengths spanning from 1300 to 1660 nm. The *x*–*y* cross sections are shown at the target focal plane for each wavelength. **d** Corresponding experimental results for metalens M1B, composed of Generation 1B meta-units, showing substantially suppressed parasitic focal spots, little elongation of the depth of focus as observed in **c**, and larger operational bandwidth

To explore the impact of meta-unit libraries on metalens performance, we fabricated and characterized metalenses using all three generations of libraries (the parameters of the fabricated metalenses are summarized in Supplementary Information Table S1). We fabricated these metalenses using a standard electron beam lithography, lift-off, and etch procedure (Materials and methods section). Scanning electron microscope images of two sample fabricated metalenses are shown in Fig. 2g, h. Using the custom-built setup shown in Fig. 3a, we characterized the three-dimensional (3D) intensity distribution of light exiting the fabricated metalenses.

Using the Generation 1A library, we realized an achromatic metalens, M1A, with a diameter of 100 µm, designed focal length of 200 µm (NA ≈ 0.24), and operational bandwidth of *λ* = 1300–1650 nm. The measured intensity distributions in the focal (*x*–*y* cross section) and axial (*x*–*z* cross section) planes at different wavelengths for M1A are plotted in Fig. 3c. From the axial intensity distributions, we can see that the chromatic aberration is significantly reduced across the entire operating bandwidth, with the focal planes for all wavelengths lying very close to one another. It is important to stress that this is a continuous aberration correction over the entire designed wavelength range, not just at the selected wavelengths. However, we can see parasitic focal spots for wavelengths shorter than 1400 nm and an elongation of the depth of focus (DOF) for wavelengths longer than 1450 nm. We attribute these features to the limited coverage of the phase-dispersion space provided by the Generation 1A library (Fig. 2d) for this lens.

**Fig. 4:**
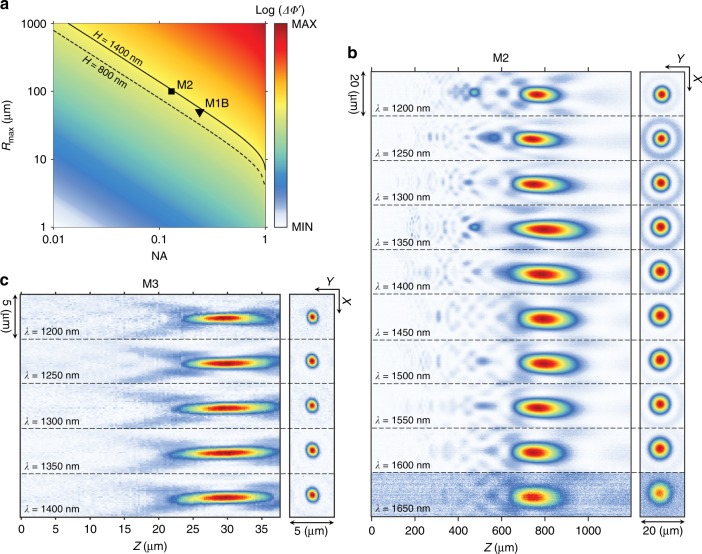
Exploring the fundamental limitations in designing achromatic metalenses. **a** Diagram showing that the range of dispersion, ${\mathrm{\Delta \Phi }}^\prime = \left( {\left. {\frac{{{\mathrm{d}}\phi }}{{{\mathrm{d}}\omega }}} \right|_{{\mathrm{max}}} - \left. {\frac{{{\mathrm{d}}\phi }}{{{\mathrm{d}}\omega }}} \right|_{{\mathrm{min}}}} \right){\mathrm{\Delta }}\omega$, achieved by a meta-unit library sets an upper limit on the achievable combinations of NA and radius, *R*_max_. In particular, the solid and dashed curves represent the limitations on achievable metalenses using the Generation 1B and 1A libraries, respectively. Two demonstrated metalenses M1B and M2, indicated in the diagram by triangle and square symbols, respectively, reach the fundamental limits. **b** Measured far-field intensity distributions for metalens M2 (*D* = 200 μm and NA = 0.12), implemented with the Generation 1B library. **c** Measured far-field intensity distributions for metalens M3 (*D* = 100 μm and NA = 0.85), implemented with the Generation 2 library

We therefore designed and fabricated metalens M1B, with the same size and NA as M1A, using the taller meta-units from the Generation 1B library. The corresponding measured focal and axial intensity distributions are plotted in Fig. 3d and show substantially suppressed parasitic focal spots and little elongation of the DOF. The wavelength range over which the chromatic aberration is corrected is also expanded to *λ* = 1200–1650 nm, a 100-nm improvement over the operational bandwidth of M1A. The combination of size, NA, and bandwidth of M1B reaches the fundamental limitation described by Eq. 5 (Fig. 4a). The predicted benefits (Supplementary Information Figure S4) of expanded coverage of the phase-dispersion space are clearly demonstrated, despite the increased challenges involved in the fabrication. We believe that this is the best reported result for a broadband polarization-independent achromatic metalens working in transmission mode.

Using the Generation 1B library, we created metalens M2, with a diameter of 200 µm and a focal length of 800 µm (NA ≈ 0.13), with chromatic aberration correction over the same wavelength range of 1200–1650 nm (Fig. 4b). This larger lens (marked by a black square in Fig. 4a) still falls within the limits set by the phase-dispersion coverage of our Generation 1B library and represents an alternative choice in the tradeoff between metalens size and NA described by Eq. 5.

Increasing the NA of metalenses comes at a cost of the operational bandwidth. We used the Generation 2 library and demonstrated a high-NA metalens M3, with a diameter of 100 µm and a focal length of 30 µm (NA ≈ 0.88), with chromatic aberration correction over a reduced operational bandwidth of *λ* = 1200–1400 nm (Fig. 4c).

**Fig. 5:**
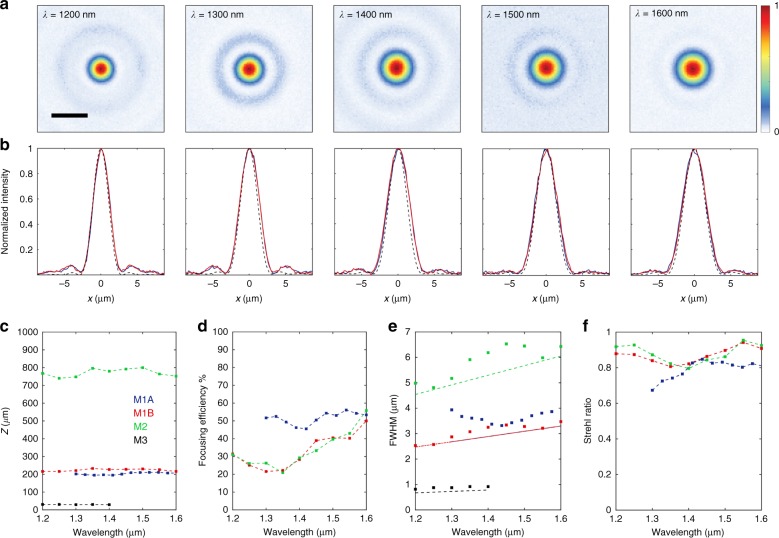
Measured figures of merit of the metalenses. **a** Focal plane intensity distributions of metalens M1B (*D* = 100 μm and NA = 0.24) at select wavelengths. Scale bar: 5 μm. **b** Horizontal (blue curves) and vertical (red curves) cuts across the measured focal spots in **a** compared with an ideal Airy spot (black dashed curves). **c** Focal lengths as a function of wavelength for the four metalenses demonstrated. **d** Measured focusing efficiencies of the metalenses, excluding M3 because its NA exceeds that of the measurement setup. **e** Extracted FWHM of focal spots for the four metalenses. Straight lines represent the theoretical FWHM. **f** Calculated Strehl ratios for the metalenses, excluding M3 because Strehl ratio measurements are not applicable for high-NA lenses.

Figure 5 summarizes important figures of merit, including the focal length, focusing efficiency, focal spot size, and Strehl ratio, for all of the fabricated metalenses. Figure 5a shows focal plane intensity profiles at the selected wavelengths shown in Fig. 3d for metalens M1B; Fig. 5b shows corresponding horizontal and vertical cuts of the measured focal spots with an ideal Airy disk overlaid for comparison. The results show nearly diffraction-limited focal spots for all wavelengths with no obvious distortion.

Figure 5c shows the measured focal lengths of the metalenses at sampled wavelengths, with the maximum shift from the mean focal length limited to 2–5% for the entire design bandwidth. Measured focusing efficiencies, defined as the percentage of power incident on the metalens and transmitted through a circular aperture positioned on the focal plane of the metalens with a radius equal to a few times the full-width at half-maximum (FWHM) of the focal spot, are plotted in Fig. 5d.

The efficiencies of our metalenses are not as high as those of the best monochromatic metalenses based on dielectric resonators^35–38^ but are significantly higher than those of metalenses based on plasmonic scatterers^8,30,31,45,48^ and recent reports of dielectric achromatic metalenses^46,47,49,50^. In all cases, sources of reduced efficiencies in achromatic metalenses include amplitude variations (not all meta-units have the same scattering efficiency), phase errors due to mismatch between the required and actual phase responses of the meta-units, nearest-neighbor effects (the optical response of a meta-unit is perturbed by adjacent meta-units, more pronounced for the taller library due to the increased interaction length), and fabrication errors (such as sidewall roughness and slope). In our transmission-mode metalenses, backscattering from meta-units can further reduce the overall efficiency. Another symptom of these errors is the presence of multiple parasitic focal spots, appearing in varying degrees in the fabricated devices but with significantly smaller intensity than the primary focal spots. These spots are commonly seen in recent achromatic metalens attempts, and the present results are of comparable magnitude to previous efforts.

Diffraction-limited focal spots are important for high-performance imaging systems. We measured the FWHM of the focal spots of all of the metalenses at sampled wavelengths and compared them with the theoretical limit $\left( {\sim \frac{\lambda }{{2{\mathrm{NA}}}}} \right)$. The results are plotted in Fig. 5e and show that for all of the lenses based on the 1400-nm library, the focal spots are at or near the diffraction limit for the entire operational wavelength range of the lenses. The focal spots for M1A, which is based on the 800-nm library, are slightly larger than the diffraction limit for some wavelengths. The performance can be further quantified by measuring the Strehl ratio^39,40^ of the focal spots. The calculated Strehl ratios for the entire bandwidth of metalenses M1A, M1B, and M2 are plotted in Fig. 5f; the ratios are above 0.8 for M1B and M2 for all wavelengths, satisfying the condition for diffraction-limited focal spots.

## Discussion

We conclude with a discussion on the utility of the present work for compact imaging systems. We believe that a combination of the present framework with the approach of recent work on achieving wide-angle monochromatic imaging^40,41^ can simultaneously reduce or eliminate chromatic and monochromatic aberrations, fully realizing the promise of metalenses. Our approach is equally capable of realizing diverging lenses (Supplementary Information Figure S8), which are also utilized in imaging systems. Additionally, a combination of several metalenses can substantially improve achromatic performance past the theoretical limitation of a singlet. For instance, stacking *N* achromatic metalenses would decrease the achievable focal length for the same diameter, bandwidth, and meta-unit library by approximately the same factor *N* (Supplementary Information Section VIII and Figure S6).

Singlet metalenses merit consideration as stand-alone devices as well, despite their well-known off-axis aberrations. A singlet achromatic metalens is suitable for broadband collimation in a flat form-factor (a collimator does not have to operate off-axis). Such a metalens could be packaged on the end of fiber optics for broadband and compact functionality^54,55^. Additionally, high-NA microlens arrays are of considerable interest for applications such as light-field cameras and CMOS camera sensors.

We note that the present approach is directly applicable to other optical frequencies with a change of material platform. For instance, visible metalenses could be realized using TiO_2_ on quartz^37,38,46,50^ or GaN on sapphire^49^. The magnitude of the refractive index, intrinsic material dispersion, and aspect ratio achievable in nanofabrication are important considerations for choosing a platform. A larger refractive index enables an increased diversity of optical response from structural dispersion engineering, and intrinsic material dispersion can add to structural dispersion to improve coverage of the phase-dispersion space. For a given dielectric material, the ultimate limiting factor on metalens performance using the present approach is the height of the meta-units, which sets the maximum range of phase dispersion. Without incorporating plasmonic or Mie resonance or meta-units with anomalous dispersion, we believe that the meta-unit libraries presented here fully utilize the phase-dispersion variety within a given height (Supplementary Information Section IX and Figure S7). For crystalline silicon, very high aspect-ratio structures are achievable^56^, motivating future work to improve on the performance demonstrated here.

In conclusion, we have demonstrated continuous diffraction-limited achromatic focusing across a broad near-infrared bandwidth. In particular, our metalenses work in the transmission mode with incident light of any arbitrary polarization state, which is highly desirable for shrinking imaging systems. We introduced a clarifying framework for understanding the limitations on chromatic aberration correction and the tradeoffs therein and have developed an approach that jointly utilizes the spectral degrees of freedom in the lens phase profiles and the geometric degrees of freedom in the meta-units to create achromatic metalenses reaching these limits. We describe and employ a phase-dispersion space that proves integral to framing and solving this problem. Our experimental results validate this approach, and these new metalenses offer a novel method for achieving chromatic aberration correction across a continuous and broad bandwidth in compact imaging systems.

## Materials and methods

### Error minimization during metalens design

The spectral degree of freedom, *C*(ω), is determined by the parameters *r*_0_ and *C*_0_, which can be used to best match a target metalens phase function with the available meta-unit library. To select the optimal meta-unit at each lattice point (*x*,*y*) of a metalens, we devise an algorithm that sweeps over possible combinations of *r*_0_ and *C*_0_ and determines the best possible set of meta-units for the metalens.

The figure of merit, FoM, used is the summation of phasor errors across all design wavelengths and lattice positions. The target phasor of a metalens is $A_{\mathrm{t}}e^{i\phi _t\left( {x,y,\lambda _j} \right)}$, where *A*_t_ is a target amplitude (which is ideally set to 1, but in practice is set according to a typical transmittance value for the given meta-unit library, e.g., 0.75) and $\phi _{\mathrm{t}}\left( {x,y,\lambda _j|r_0,C_0} \right)$ is the phase profile of the metalens given a value of *r*_0_ and *C*_0_. Each meta-unit has a simulated phasor $A_{\mathrm{m}}\left( {\lambda _j} \right)e^{i\phi _m\left( {\lambda _j} \right)}$ stored in the meta-unit library, and the difference (error) between the target phasor and the meta-unit phasor is calculated at each (*x*,*y,λ*_*j*_). The meta-unit with the minimal error summed across the wavelengths is recorded as the best choice for that (*x*,*y*). The error of each optimal choice is summed across all points (*x*,*y*) and recorded as the FoM for that *r*_0_ and *C*_0_. The values of *r*_0_ and *C*_0_ corresponding to the smallest FoM are then chosen.

This error minimization algorithm minimizes errors for a specific set of discrete wavelengths (in practice, we select many closely spaced wavelengths, approximating a continuous spectrum). The algorithm takes into account deviations from the linear phase response presumed under the phase-dispersion framework (which only strictly considers the lowest and highest frequencies and linearly interpolates the phase in between) and straightforwardly includes simulated amplitude deviations from unity (variations in amplitude across a metasurface lead to high diffraction orders).

### Metalens fabrication

Amorphous silicon films of thickness 800 or 1400 nm are deposited by plasma-enhanced chemical vapor deposition at 200 °C on 500-μm fused quartz substrates. A JEOL JBX-6300FS electron beam lithography apparatus is used to define the metasurface lens pattern on a double-layer resist (PMMA 495k A4 and 950A2) with a dose of 770 μC/cm^2^ at a current of 200 pA. A 20-nm layer of E-Spacer is spun on top of the double-layer resist to avoid the electron charging effect. After the resist is developed in IPA:DI (3:1) for 2 min at 5 °C, 20 nm of aluminum oxide is deposited using electron beam evaporation and lifted off in Remover 1165. The metasurface lens pattern is transferred to amorphous silicon films by inductively coupled plasma (ICP) etching in a mixture of SF_6_ (40 sccm) and O_2_ (16 sccm) at −100 °C with an ICP power of 800 W and an RF power of 40 W.

### Optical characterization

In the experimental setup (Fig. 3a), a collimated laser beam with a tunable wavelength is incident on the metalens, and the 3D far-field of the lens is measured by acquiring a stack of 2D images at different distances from the lens. The tunable laser beam is generated by passing the emission from a supercontinuum laser source (NKT SuperK Extreme) through a monochromator (Horiba iHR550). Light exiting the monochromator is coupled into a single-mode fiber (Thorlabs P1-980A-FC) and then collimated using an adjustable aspheric fiber collimator (Thorlabs CFC-5X-B) with an anti-reflection coating for a wavelength range of 1050–1620 nm. The imaging optics for monitoring the far-field of the metalenses consist of a ×100 objective (Mitutoyo Plan Apo NIR), a tube lens (Thorlabs AC254-200-C) of focal length 200 mm, and an InGaAs camera (Princeton Instruments Nirvana 640ST), which are mounted on a motorized stage.

Using the stage, the device plane is brought to focus on the camera. Then, the stage is moved toward the focal plane of the metalens in predefined increments (typically 1 μm). At each position, the transverse intensity distribution is captured by the camera for desired wavelengths by changing the angle of the grating in the monochromator. In this way, the 3D far-field of the metalens is measured. The longitudinal intensity distribution is then created for each wavelength by splicing the 3D intensity distribution along the axis of the metalens.

For efficiency measurements of the metalenses, a flip mirror and an iris are introduced before the InGaAs camera. The power transmitted through a metalens is measured by focusing the camera on the device plane and closing the iris to match the diameter of the metalens. The power is then passed to a power meter (Thorlabs SM05PD5A) using the flip mirror and a lens with a 40-mm focal length. The incident power is measured by focusing the camera on an unpatterned region of the quartz substrate and recording the power with the iris set to the same diameter as the metalens. The ratio between transmitted and incident power defines the transmission efficiency. To measure the focusing efficiency, the motor is first moved to the focal plane of the metalens and the iris is closed such that it corresponds to three to five times the FWHM of the focal spot. Then, the light is passed to the power meter. The ratio between the measured focused power and the incident power defines the focusing efficiency.

To determine any chromatic aberration in our imaging optics, we image gold nanostructures at different wavelengths by bringing them to focus using the motorized stage and recording the position of the stage for each wavelength. In this way, we ascertain the chromatic aberration of our imaging optics; this information is used to correct the 3D intensity measurements of the metalenses.

## Electronic supplementary material

Supplementary Materials
