# Supplementary material for: Broadband achromatic dielectric metalenses
Source: Light Sci Appl. 2018 Nov 7;7:85. doi: 10.1038/s41377-018-0078-x (PMC6220161; doi:10.1038/s41377-018-0078-x)
Supplement: Supplementary file 1 — Supplementary Materials [file 41377_2018_78_MOESM1_ESM.docx]

Supplementary Information

**Broadband Achromatic Dielectric Metalenses**

Sajan Shrestha^1,#^, Adam C. Overvig^1,#^, Ming Lu^2^, Aaron Stein^2^, and Nanfang Yu^1,^*

^1^Department of Applied Physics and Applied Mathematics, Columbia University, New York City, New York, 10027, United States
^2^Brookhaven National Laboratory, Center for Functional Nanomaterials, Upton, New York, 11973, United States

*^#^*These authors contributed equally to this work.

**I. Geometric interpretation of the spectral degree of freedom**

A typical approach to derive the required phase function of a metasurface involves the holographic principle. In the case of a metalens, this corresponds to placing a dipole source at the focal spot and propagating the emitted spherical wave to the plane of the metasurface. This process can be visualized as in Fig. S1a, wherein a black semi-circle represents a reference wavefront of the dipole centered at the focal point, $f$. The phase function is related to the segment, $d$, of ray $\rho$, spanning from position $r$ to the semi-circle,

$$d=\sqrt{r^{2}+f^{2}}-f.$$

It is apparent that a phase *advance* (a negative value) proportional to the propagation phase across a distance $d$ must be added at each position $r$. That is, at position $r$ the hemispherical wavefront must jump *ahead* a distance $d$ in order to match the planar metasurface. The phase function is therefore easily written down as:

$$\phi\left( r,\omega\right)= -k_{0}d= -\frac{\omega}{c}\left( \sqrt{r^{2}+f^{2}}-f \right),$$

which is recognizable as the conventional form of the phase function. The natural dispersion of this form is seen by the dashed red, green, and blue wavefronts in Fig. S1a. The wavefronts for each color overlap at the reference wavefront, but diverge due to dispersion on either side. The sign of this dispersion is opposite above and below the reference wavefront.

If we instead choose to propagate the reference wavefront by a distance $\sqrt{r_{0}^{2}+f^{2}}$, it will intersect the plane of the metalens at a position $r_{0}$. This choice is depicted in Fig. S1b, wherein two zones of the metalens are thereby defined: for $r>r_{0}$, the sign of the dispersion at the metalens is the same as the conventional choice, but for $r<r_{0}$, it is opposite. In this region, the hemispherical incident wavefront must jump backwards to meet the metalens, corresponding to a phase delay that is proportional to a distance $d$. The phase function in this case is again easily written down as;

$$\phi\left( r,\omega\right)= -k_{0}d= -\frac{\omega}{c}\left( \sqrt{r^{2}+f^{2}}-\sqrt{r_{0}^{2}+f^{2}} \right).$$

b

a


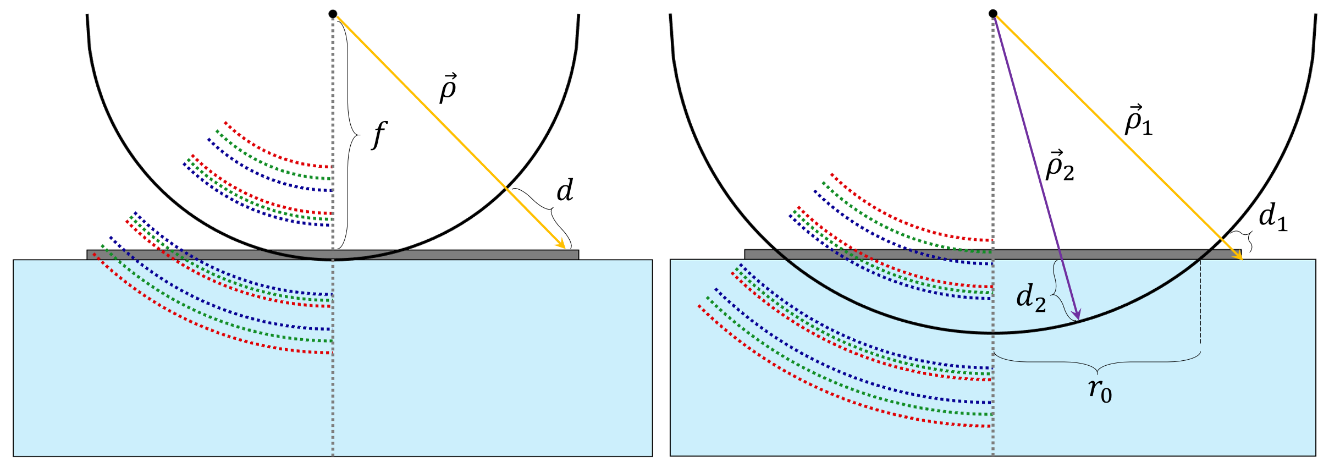


**Fig. S1 Geometrical interpretation of** $C(\omega)$**, the spectral degree of freedom. a,** Schematic of the conventional geometrical derivation of the phase function of a metalens. A reference wavefront (black) defines a segment $d$ of ray $\vec{\rho}$ representing the distance an incident planewave must jump to meet the converging wavefront. Chromatic wavefronts diverge with opposite signs on either side of this reference wavefront, and can be seen intersecting the metalens in the order Blue, Green, Red for increasing distance from the central axis. **b,** Schematic of the proposed geometrical derivation of the phase function of a metalens. The reference wavefront is extended past the surface of the metalens; chromatic wavefronts can be seen intersecting the metalens in the order Red, Green, Blue for increasing distance from the central axis up to the radius $r_{0}$.

It is apparent from comparison of Fig S1a with Fig S1b that the sign of the dispersion of the chromatic wavefronts (i.e., intersection of red, green, and blue wavefronts with the metasurface) is opposite near the center of the lens. The location of the reference wavefront (determined by $C(\omega))$ therefore determines the sign of the dispersion required by the metalens.

Finally, we clarify the separate role $C_{0}$ plays. Generalizing the phase function above to include $C_{0}$, we can write:

$$\phi\left( r,\omega\right)= -\frac{\omega}{c}\left( \sqrt{r^{2}+f^{2}}-\sqrt{r_{0}^{2}+f^{2}} \right)+C_{0}.$$

It is readily apparent that $\phi\left( r=r_{0},\omega\right)=C_{0}$ is independent of frequency. Therefore, $C_{0}$ is the value of the phase of the reference wavefront, and $r_{0}$ is the position of that wavefront. In a monochromatic metalens, $r_{0}$ and $C_{0}$ play a duplicate role, and only in achromatic metalenses does the distinction become relevant. In this way, the reference position $r_{0}$ can be interpreted as extending the concept of reference phase to include reference dispersion for the case of broadband metalenses.

**II. Derivation of proposed** $\boldsymbol{C(\omega}\mathbf{)}$ **from Generalized Snell’s Law**

Deriving the form of the phase function of a metalens from the Generalized Snell’s Law, or conservation of momentum, is a brief yet clarifying exercise. We present it here to motivate the proposed form of $C(\omega)$ from first principles. We begin with the observation that a converging metalens needs to deflect an incoming planewave by an angle $\theta$ at position $r$ corresponding to:

$$\sin\left( \theta\right)=\frac{r}{\sqrt{r^{2}+f^{2}}}.$$

By the Generalized Snell’s Law, this corresponds to a phase gradient:

$$\frac{d\phi\left( r,\omega\right)}{dr}=k_{0}\sin\left( \theta\right)=\frac{\omega}{c}\frac{r}{\sqrt{r^{2}+f^{2}}},$$

where $k_{0}=\frac{\omega}{c}$ is the free-space wavevector (we take the metalens to be focusing in air). Indefinite integration of the above leads to the phase function,

$$\phi\left( r,\omega\right)= \int dr\frac{\omega}{c}\frac{r}{\sqrt{r^{2}+f^{2}}}= -\frac{\omega}{c}\sqrt{r^{2}+f^{2}}+C(\omega),$$

and the Fundamental Theorem of Calculus obtains it in definite form,

$$\phi\left( r,\omega\right)= \int_{r_{0}}^{r} dr'\frac{\omega}{c}\frac{r'}{\sqrt{{r'}^{2}+f^{2}}}= -\frac{\omega}{c}\left\{ \sqrt{r^{2}+f^{2}}-\sqrt{r_{0}^{2}+f^{2}} \right\},$$

where $r_{0}$ is a reference radius where the integration begins. A comparison to the indefinite form shows the choice:

$$C\left( \omega\right)=\frac{\omega}{c}\sqrt{r_{0}^{2}+f^{2}},$$

which is identical to equation (2) in the main text with the particular choice $C_{0}=0$.

The spectral degree of freedom $C\left( \omega\right)$ is therefore understood to be the same degree of freedom inherent to integration. The indefinite form puts no limitations on the form of $C(\omega)$, allowing it to be any function of frequency, including nonlinear functions of $\omega$. Since both a typical meta-unit response as well as propagation phase have a linear dependence on frequency, a linear form of $C(\omega)$ is the natural choice. With the addition of the $C_{0}$ to reflect the arbitrary choice of the value of the reference phase, the definite form of the integral provides the most general linear form of $C(\omega)$ while abiding the natural physical interpretation explored in Supplementary Section 1.

**III. Derivation of lens limitations**

An understanding of the relationship between desired lens parameters (the numerical aperture, *NA*, and the lens diameter, $D=2R$, metasurface lattice spacing *P*) and the resulting required area of phase-dispersion space is important.

The first consideration is in the phase sampling rate of the metasurface, which is constrained by the largest deflection angle for the smallest wavelength. This constraint can be made rigorous by considering the magnitude of the rate of phase change as a function of radial position:

$$\left| \frac{d\phi}{dr} \right|=\frac{\omega}{c}\frac{r}{\sqrt{f^{2}+r^{2}}}. \left( S1 \right)$$

Using the definition of *NA*:

$$NA=n\sin\left( \theta\right)=n\frac{R}{\sqrt{f^{2}+R^{2}}}, (S2)$$

then for a lens operating in air ($n=1)$, Eq. S1 at $r=R$ for the smallest wavelength simplifies to

$$\left| \frac{d\phi}{dr} \right|_{r=R}=\frac{\omega_{max}}{c}NA=k_{max}NA. (S3)$$

Requiring at least $N$ phase samples in a $2\pi$ phase range in a metasurface with periodicity $P$ amounts to the constraint:

$$\left| \frac{d\phi}{dr} \right|_{r=R}<\frac{2\pi}{NP}. (S4)$$

The choice of $N$ will impact the efficiency of the metalens, with higher values of $N$ producing higher efficiencies of diffraction into the 0^th^ order. Combining equations (S3) and (S4) gives the constraint of:

$$NA<\frac{\lambda_{min}}{NP}. (S5)$$

Equation (S5) describes the tradeoff between the $NA$ of a lens and its bandwidth of achromatic performance. The lower bound wavelength is directly represented as $\lambda_{min}$, while the upper bound is implicitly represented in $P$. If $P$ is too small compared to a desired $\lambda_{max}$, the flexibility of structural dispersion engineering within a meta-unit area of $P$×$P$ is restrained (i.e., phase-dispersion space is more difficult to fill). Thus equation (S5) clarifies the impact of meta-unit library design on achievable lens performance.

The second consideration is that the limitations introduced by the finite coverage of the meta-unit library in phase-dispersion space. For simplicity, we focus on a meta-unit library that spans a finite range along the dispersion axis ($\Delta\Phi^{'}=\left( \left. \frac{d\phi}{d\omega} \right|_{\max}-\left. \frac{d\phi}{d\omega} \right|_{\min} \right)\Delta\omega)$ while fully spanning the phase axis throughout this range. Since the extremes of dispersion are represented at the center and edge of the metalens, the required range of dispersion can be written:

$$\frac{d\phi\left( 0,\omega\right)}{d\omega}-\frac{d\phi\left( R,\omega\right)}{d\omega}=\frac{d}{d\omega}\left( \frac{\omega}{c}\sqrt{f^{2}+R^{2}}-\frac{\omega}{c}\sqrt{f^{2}} \right). (S6)$$

The limitations of a finite $\Delta\Phi^{'}$ can therefore be written:

$$\Delta\Phi^{'}\geq\frac{\Delta\omega}{c}\left( \sqrt{f^{2}+R^{2}}-\sqrt{f^{2}} \right). (S7)$$

Using equation (S2) again with $n=1$, we arrive at a relationship between the maximum achievable radius of the metalens $R_{max}$, $NA$, operational bandwidth $\Delta\omega$, and the characteristic span $\Delta\Phi^{'}$ along the dispersion axis:

$$R_{max}\leq\frac{\Delta\Phi^{'} c}{\Delta\omega\left( \frac{1}{NA}-\sqrt{\frac{1}{NA^{2}}-1} \right)}\boldsymbol{,}(S8)$$

which simplifies to $R_{max}\leq\frac{2\Delta\Phi^{'} c}{\Delta\omega NA}$, when *NA*<<1.

IV. Structural dispersion in dielectric meta-units

In order to fill as much of phase-dispersion space as possible, the dielectric meta-unit library must contain a diverse set of phase and dispersion responses. Within the model of dielectric meta-units as vertical waveguides this suggests structuring the cross-section of the meta-units in order to get as diverse a set of effective index dispersion as possible. Figure S.2 depicts structural dispersion in five example meta-units, chosen to represent various regions of phase-dispersion space. In the waveguide model, the phase is approximately:

$$\phi_{0}=\frac{\omega_{min}}{c}n_{eff}\left( \omega_{min} \right)H,$$

while the dispersion is approximately

$$\Delta\phi=\frac{H}{c}\left( n_{eff}\left( \omega_{max} \right)\omega_{max}-n_{eff}\left( \omega_{min} \right)\omega_{min} \right),$$

from which the role of the dispersion of $n_{eff}\left( \omega\right)$ is clarified. If $n_{eff}\left( \omega\right)$ is high for all frequencies, both the phase and dispersion will be large; if it is low for all frequencies, both the phase and dispersion will be low; if it is high for the highest frequency, but low for the lowest frequency, the phase will be low but the dispersion will be high. Several such example index profiles are seen in Fig. S2b, with the field profiles visualized in Fig. S2a and the corresponding points in phase-dispersion space marked in Fig S2c.


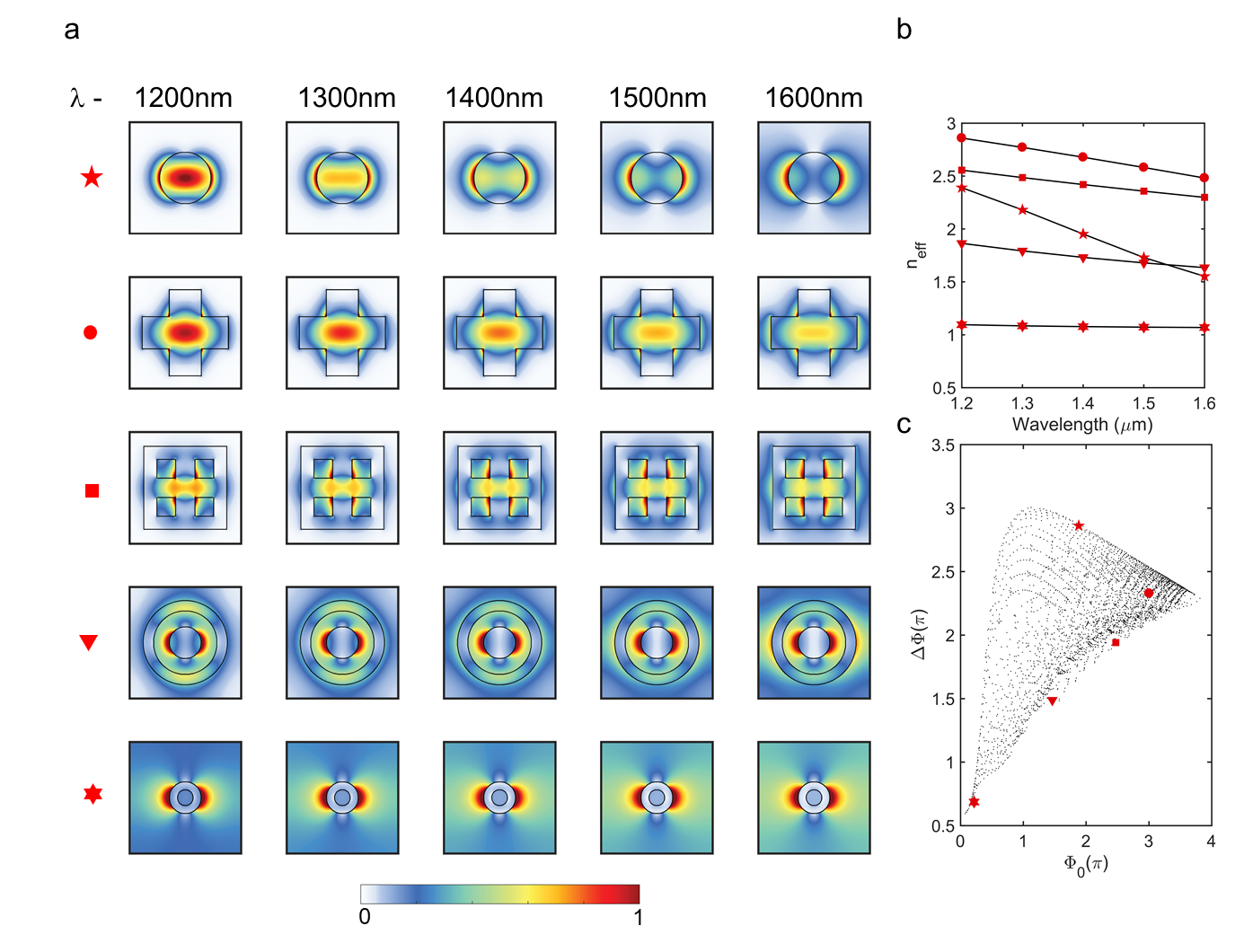
**Fig. S2 Mode profiles of example meta-units.** **a,** Each row corresponds to a single meta-unit, and each column a single wavelength. The first row depicts the most dispersive meta-unit, showing that for the lowest wavelength of 1,200 nm, the modal overlap with silicon is very high; conversely, for the longest wavelength of 1,600 nm, the modal overlap with silicon is quite low. This makes the effective index highly dispersive. In contrast, from top to bottom, each row depicts a meta-unit with successively smaller dispersion. For instance, in the final row the mode profiles of the shortest and longest wavelength are similar, implying little structural dispersion. **b,** Corresponding effective indices of the meta-units. **c,** Location of the meta-units in phase-dispersion space overlaid on top of a representative meta-unit library.

While calculating the phase response of our meta-units, we ignored the possibility of power coupling into higher-order waveguide modes. We can estimate the correct effective index of each meta-unit by taking the average of effective modal indices of each propagating mode weighted by the fraction of power carried by the mode. We have numerically solved for effective modal indices of the higher-order modes and the fraction of power coupled into them from free space by using eigenmode analysis techniques (Fig. S3). This has been done for various meta-unit architypes and the error in the estimation of effective indices when ignoring the higher-order modes has been calculated (Fig. S4). From these calculations, we observe that the choice of ignoring higher-order modes does not affect the calculation of the phase response of meta-units that have small to medium cross-section, since high-order modes do not propagate in such waveguides (e.g., meta-unit disc1 and disc2, which are cylinders with radii 100 and 200 nm, respectively; see Fig. S4a). For meta-units with large cross-section in our library, up to two higher-order modes can propagate depending upon the meta-unit archetype and the wavelength (e.g., meta-unit disc3, which is a cylinder with radius 300 nm, supports two higher-order propagating modes; see Fig. S4a). We see that in the worst-case scenario of meta-units with very large cross-section, our estimation of effective indices can be off by up to 20% primarily at short wavelengths (Fig. S4c). However, these types of meta-units make up a small portion of our meta-unit library, hence our estimation of the effective indices of the meta-units based only on the fundamental waveguide modes is largely correct.


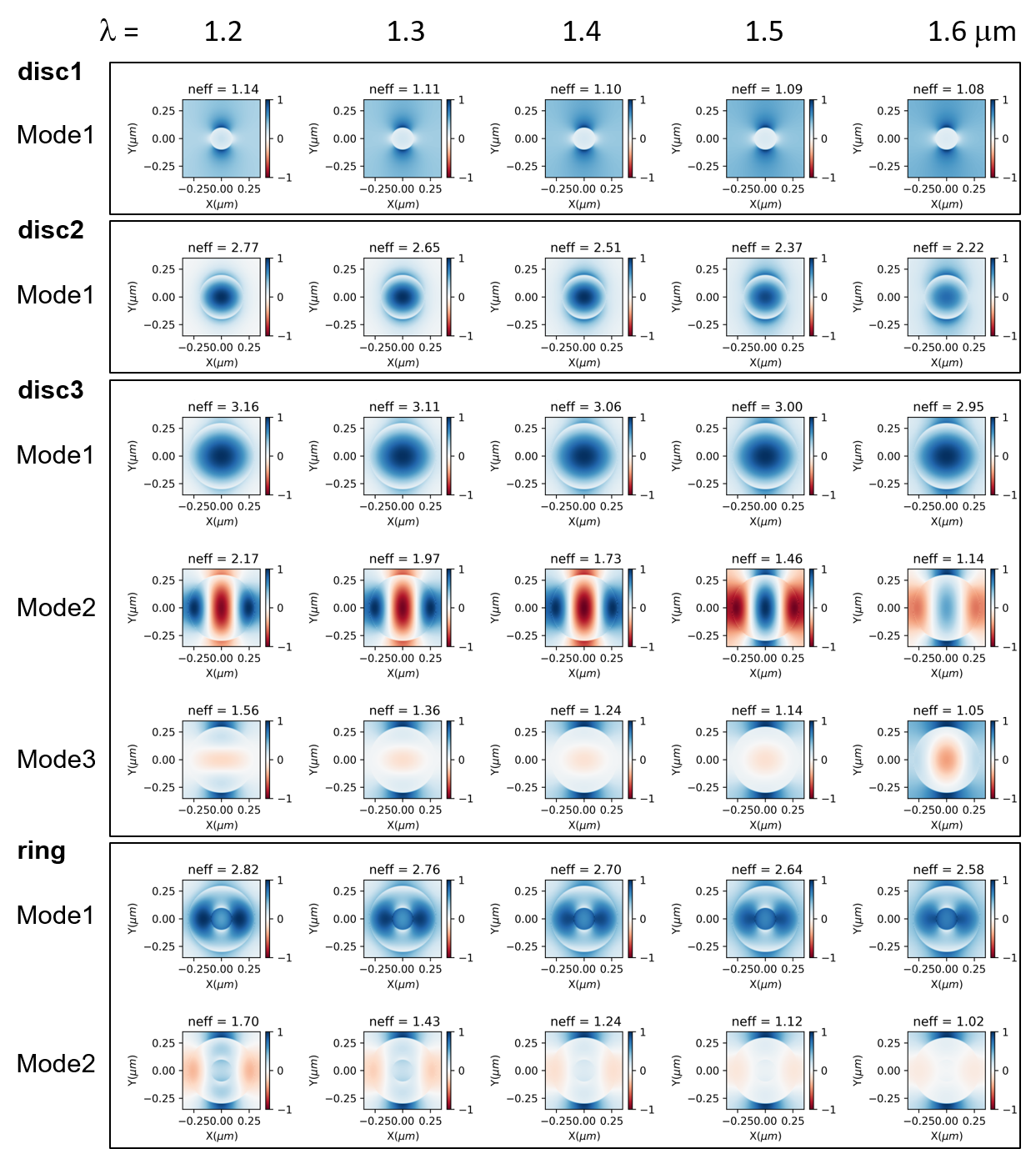


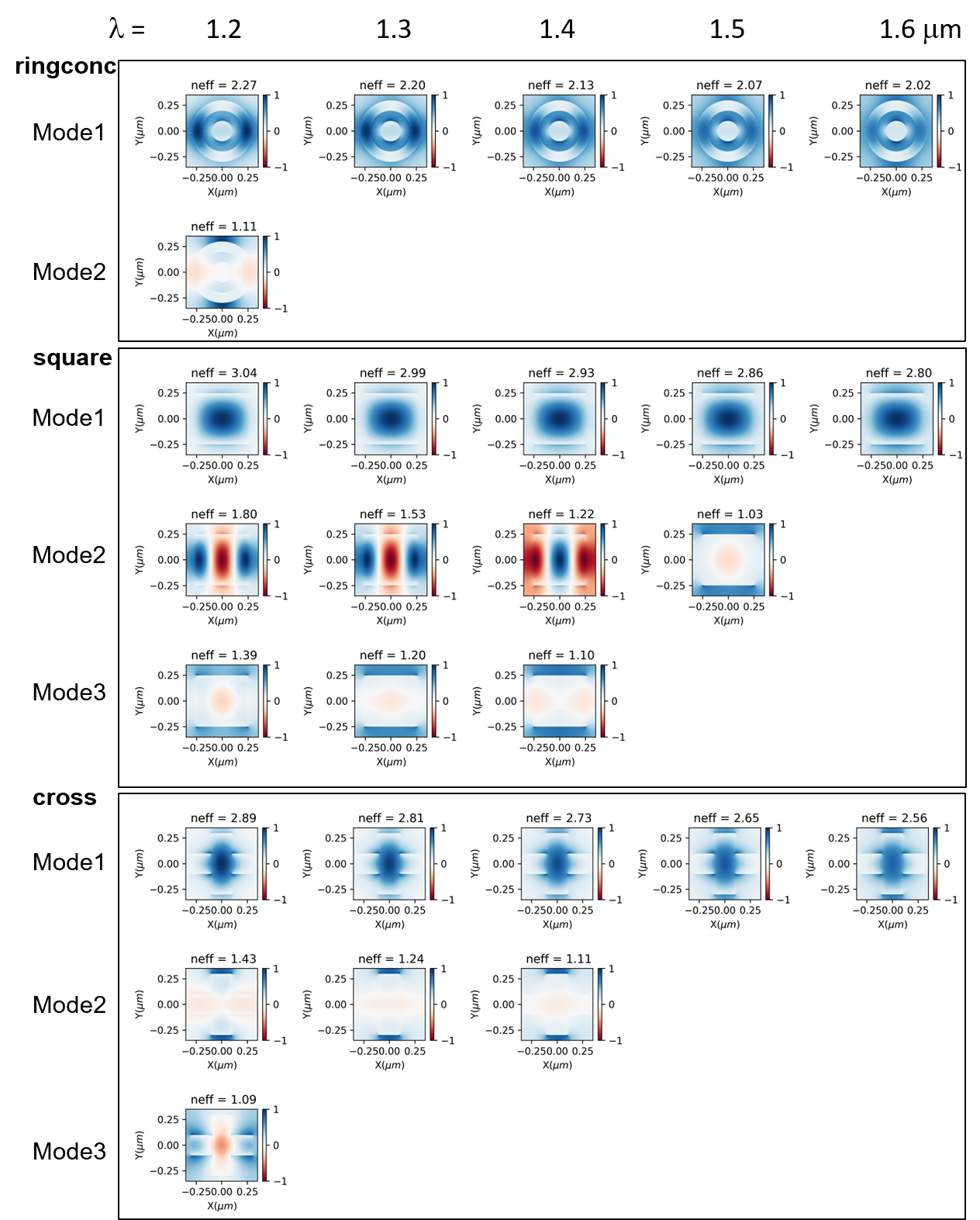


**Fig. S3 Modal analysis of meta-units.**


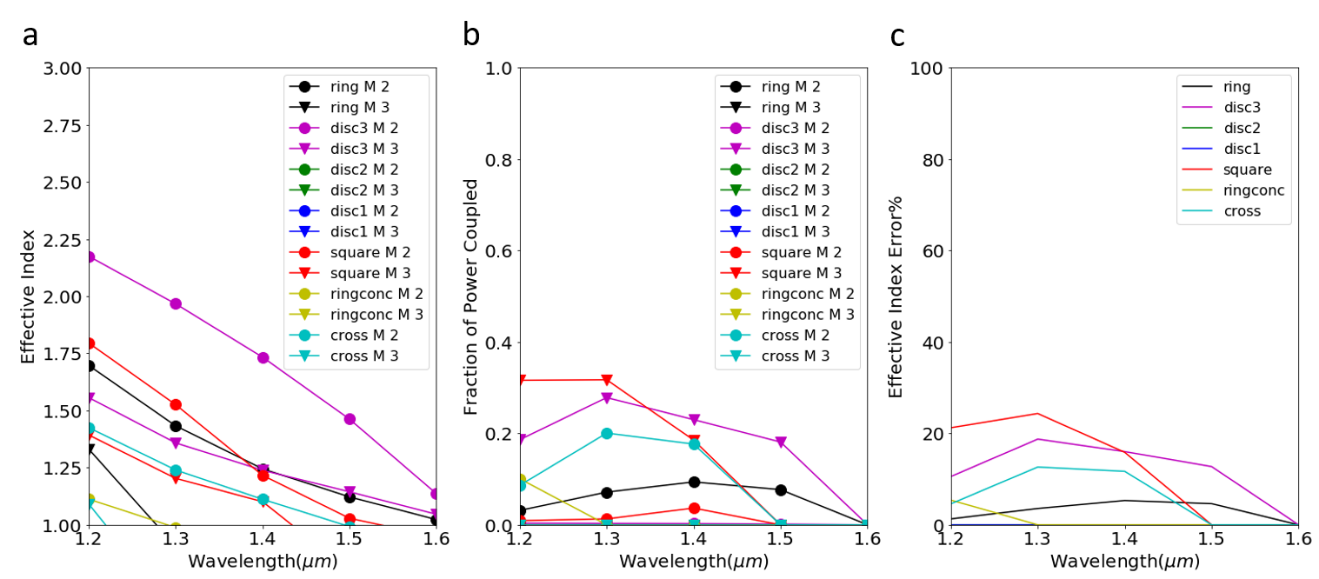


**Fig. S4 Coupling into higher-order modes.** **a,** Effective indices of the 2^nd^ and 3^rd^ order waveguide modes of selected meta-units of each archetype as a function of wavelength. Note for meta-units disc1 and disc2, which have a cylindrical cross-section of radii 100 and 200 nm, respectively, only the fundamental mode propagates. Whereas for disc3, which has a cylindrical cross-section of radius 300 nm, there are two higher-order modes (i.e., “M 2” and “M 3”) that can propagate. **b,** Fraction of power coupled into the higher-order modes. **c,** Percentage error in estimation of effective modal indices when ignoring coupling into higher-order waveguide modes.

**V. Treatment of sharp resonances of meta-units**

A notable point of divergence in the present approach compared to recent work is in the treatment of sharp Fano resonances present in infinitely periodic lattices of dielectric meta-units. These are easily calculated from simulating the meta-units in full-wave simulations (for instance, Finite-Difference Time-Domain) with periodic boundary conditions. Past efforts using reflection-based dielectric meta-unit libraries rely on these sharp resonances to provide large dispersion meta-units (the phase goes through $\sim2\pi$ across these resonances, so the dispersion is inversely proportional to the resonance bandwidth: $\frac{d\phi}{d\omega}\cong\frac{2\pi}{\Delta\omega}$).

However, we believe that there are a number of disadvantages to relying on these sharp resonances. First, this approach requires careful alignment of many such resonances. Second, narrow bandwidth resonances are by their nature difficult to reliably achieve in experiment as their long optical lifetimes intrinsically make them sensitive to small changes in structure (period, meta-unit cross-section) and surroundings. Third, they are difficult to generalize to high efficiency transmission mode metalenses, because the sharp resonances are typically associated with a transmission dip (irrelevant for reflection mode metalenses). Fourth, the resonances are born of the periodic interactions of many dielectric meta-units; a meta-unit located among dissimilar neighboring meta-units may not have the same response. This invalidates the assumption intrinsic to metasurfaces that meta-units operate approximately independently from each other. Moreover, while the periodic behavior is retained as more and more periods are included (e.g., low *NA* metalenses include regions of meta-units that are largely periodic), the degree of retention is a complex function of intrinsic optical lifetime, index contrast, and mode profile; it is difficult to reliably predict how many periods are needed. Indeed, even the bandwidth of the resonance will change as the number of periods changes, reducing the predictive power of the meta-unit library.


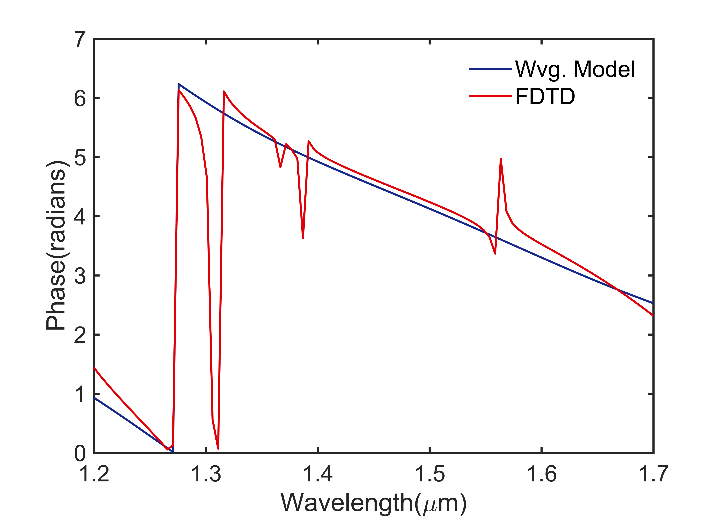


**Fig. S5 Comparison of phase responses of an example meta-unit obtained by the waveguide model and Finite-Difference Time-Domain (FDTD).** Our waveguide approximation is seen to reproduce the trend predicted by the FDTD simulation, excluding sharp resonances.

We note that while the proper treatment of sharp resonances is a subtle and open question, and submit that for broadband, high *NA* metalenses operating in *transmission* mode, the preferred approach should not rely on them. First, for rapidly changing phase profiles (especially at the edge of a metalens) the meta-units will also need to change rapidly, meaning that very few periods will be present. In these cases, the sharp spectral lines may not be realized at all. Second, even in the case that sufficient periods are present (e.g., in a region of a metalens with very slowly changing phase profile), the spectral weight of the resonances over a broad bandwidth is often very small as seen in Fig. S5. This means that while the phase/amplitude responses for a few wavelengths in this region of the metalens may be different from the prediction of the waveguide model due to the resonances, the majority of the functionality of our metalenses will be retained. Third, in our experience, many typical metalenses of interest (*NA* and radii not too small) do not have many repetitive neighboring meta-units. For such metalenses the actual behavior of a meta-unit within a metalens should be typically much closer to the waveguide approximation, which can be seen to exclude the sharp resonances in Fig. S5. The waveguide model agrees well with full-wave simulations of infinitely periodic structures away from resonances. Neither approach to constructing a meta-unit library fully captures how a meta-unit would response in a metalens, but for the above reasons we believe that the waveguide approach is a better choice.

Some resonances, however, can be quite broadband, and can result in large back-reflection due to nearest neighbor coupling of only a few similar meta-units. This particularly impacts smaller wavelengths (where more complex modal interactions are possible) while it is less relevant for longer wavelengths (where the meta-unit lattice is deeply subwavelength). The waveguide approximation does not adequately treat these meta-units because it ignores the complex modal interactions, and future work could benefit from using a combination of full-wave simulations and the waveguide approach to exclude meta-units that exhibit spectra with broadband reflection. Future work could also benefit from fully including the modal interactions of nearest neighbors in metasurface design, which is especially relevant for materials systems with refractive indices lower than that of silicon (e.g., SiN, GaN, TiO_2_).

**VI. Phase sampling of example metalens**

Figure S6a,b compare achieved phase profiles by Generation 1A and 1B libraries of a metalens with *R* = 100 μm and *NA* = 0.14. It is evident that the Generation 1B library has small phase errors across four select wavelengths at all radial positions, while the Generation 1A library has substantial phase errors at the edge of lens. This indicates that the target metalens has too large a diameter or *NA* for the Generation 1A library to satisfy.


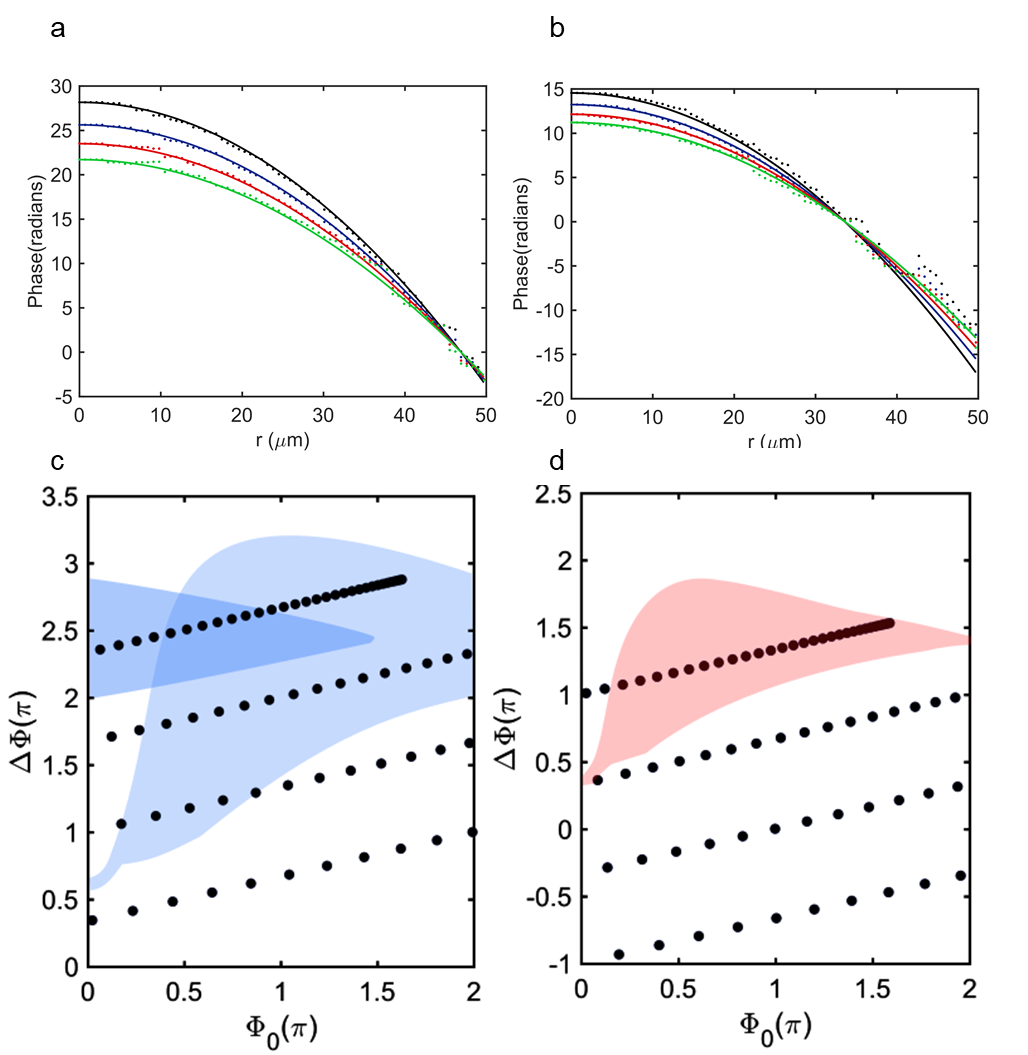


**Fig. S6 Comparison of achieved phase profiles by Generation 1A and 1B libraries of a metalens at four select wavelengths.** Black, blue, red, and green represent wavelengths of 1210 nm, 1330 nm, 1450 nm, and 1570, respectively. The metalens has *R* = 100 μm and *NA* = 0.14. Note minimization of phase errors due to improved phase coverage offered by Generation 1B library in **a**, compared to Generation 1A library in **b.**

**VII. Comparison of simulated and experimental focal spots**

In order to anchor expectations for experimental results, dipole simulations are performed using the expected responses of the meta-units retrieved from error minimization. Due to computational constraints, it is impractical to perform full-wave simulations of metalenses of the size we experimentally demonstrated. In lieu of full-wave simulations, dipole summations on a real-space grid are performed for each wavelength: a dipole is placed at each lattice point on the metalens, with wavelength dependent complex value $A(\lambda)exp(i\phi(\lambda)$), where $A(\lambda)$ is the calculated amplitude response and $\phi(\lambda)$ is the calculated phase response of the chosen meta-unit for that point. The interference of these dipole sources (indexed by $j)$ propagated towards the designed focal spot is calculated and recorded for each wavelength:

$$E\left( x,y,z,\lambda\right)= \sum_{j} A(\lambda)\exp\left( i\phi\left( \lambda\right) \right)\frac{\exp\left( i{\frac{2\pi}{\lambda}R}_{j} \right)}{R_{j}},$$

and compared to the experimental results as seen in Fig. S7.

Comparisons of this dipole approach and full-wave simulations (FDTD, Lumerical Solutions) are performed for much smaller lenses (where full-wave simulations are feasible) and the agreement is good. This suggests the validity of the dipole approach as a tool for approximate comparison to larger, experimental metalenses.


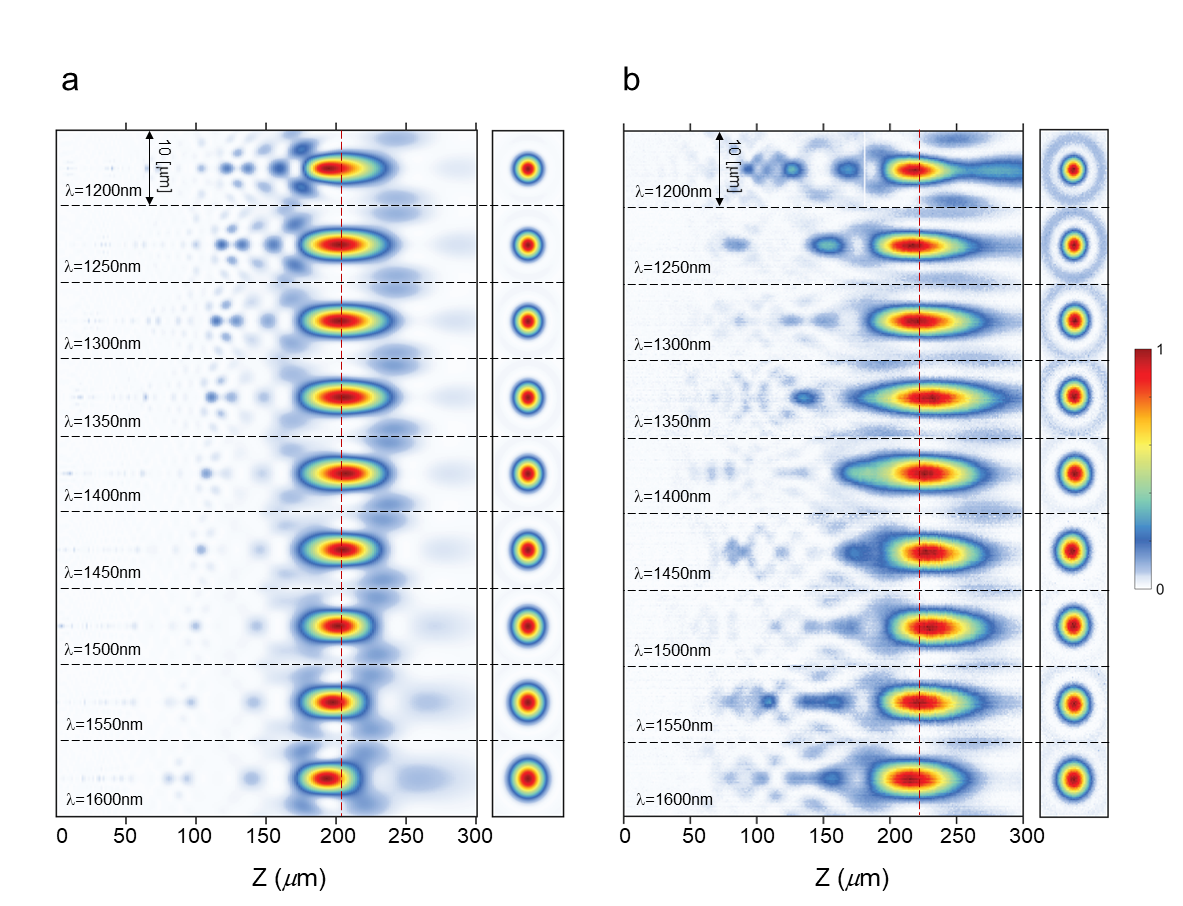


x

y

x

y

**Fig. S7 Comparison between numerical simulation and experimental results**. **a,** Dipole simulation results, showing some parasitic secondary spots, with reduced magnitude compared to experimental results in **b**.

**VIII. Simulation of compound metalens system**

As an initial exploration of the utility of the present approach for compact imaging systems based on compound metalenses, we perform dipole simulations (S.7) for an example metalens doublet. The metalenses are spaced $s=50 \mu m$ apart, and have identical parameters ($D=200 \mu m$, $f=800 \mu m)$, each of which is separately corrected for chromatic aberration.


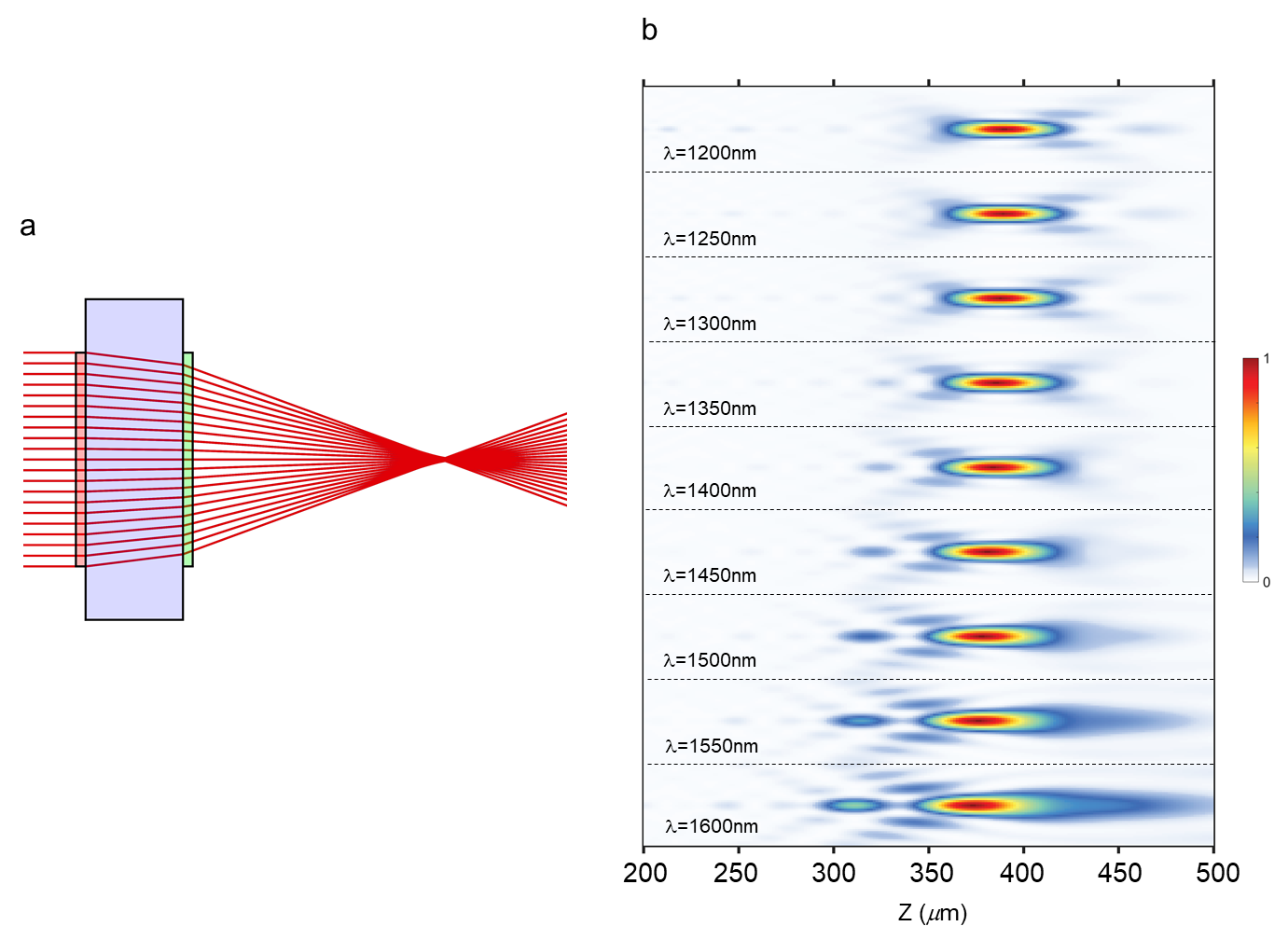
According to geometrical optics, the focal length, $F$ of the doublet can be determined by the following relation:

$$\frac{1}{F}=\frac{1}{f}+\frac{1}{f}-\frac{s}{f^{2}}.$$

The ray picture of the doublet is shown in Fig. S8a, and the dipole calculation of the far field of the metalens doublet is shown in Fig. S8b. The expected focal length agrees well with the calculated focal length. The retention of the achromatic behavior for a higher *NA* than achievable with a singlet confirms the utility of the approach for high performance compound metalens systems.

**Fig. S8 Stack of two achromatic metalenses.** **a,** Ray tracing diagram of a metalens doublet with two identical converging lenses. **b,** Axial (x-z cross-section) intensity distributions of the doublet calculated using dipole simulations where the design focal length and diameter of each metalens are 800$\mu m$ and 200$\mu m$, respectively, with spacing set to 50$\mu m$. The focal length of the doublet is calculated to be 412$\mu m$ using the paraxial thin-lens approximation.

**IX. Meta-unit library construction by binary search**

In order to determine whether the meta-unit cross-sections used fully span the phase-dispersion space for a given height, we perform a more exhaustive set of simulations. As depicted in Fig. S9a, we discretize an area of a meta-unit period into a 14×14 grid, and explore all choices of populating each pixel of this grid by either air or Silicon. To reduce the coupling between adjacent meta-units and to be consistent with our fabricated structures, we keep a boundary of air at the edges of the meta-unit period, leaving a 12×12 grid to explore. We limit the cross-sections to those with four-fold symmetry to retain polarization independent behavior. This allows the cross-sections to be defined on an octant of the grid, and leaves 21 unique pixels to define the cross-sections. The total number of simulations is therefore 2^21^ = 2,097,512.

a

b


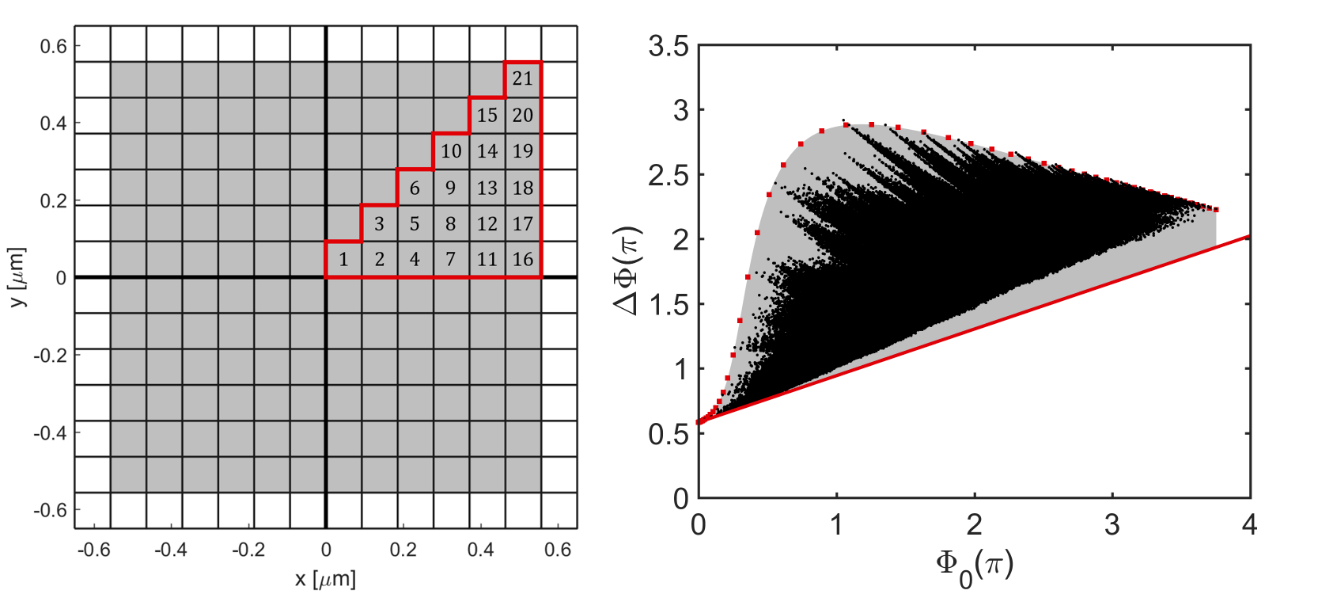


**Fig. S9** **Meta-unit library constructed from binary search. a,** Diagram showing the geometrical definition of 21 pixels that can either be air or Silicon. A boundary of air is left surrounding the modified area. The remaining choices are determined by symmetry based on the octant outlined in red. **b,** A set of 2^21^ = 2,097,152 simulations exploring all combinations of geometries. Also plotted are singular pillars (red squares) and an ideal effective medium (red line), showing that the binary search begins to fill in the shaded area bounded by these two references.

Figure S9b shows the resulting meta-unit library for a height of $H=1,400 nm$ in phase-dispersion space. Also shown are results for singular pillars as a reference, as well as an effective medium line representing the theoretical limit of performance at minimal dispersion (no structural dispersion). The area between these two references is shaded grey. It is evident that the results of this binary search begin to fill the shaded area, but do not extend past it. This suggests that the meta-unit libraries chosen based on physical intuition perform as well as the brute-force approach taken here. A finer grid would fill the shaded area better at high dispersion values, but would make the number of simulations impractical to perform.

We lastly note that the impact of the boundary of air is two-fold. First, a sharp cut-off in the shaded grey area is apparent at high phase. This represents the fact that a fully-Silicon meta-unit is never used (so the upper limit of the effective index is less than that of silicon). Secondly, as the value of phase increases, the lowest dispersion values possible begins to diverge from the effective medium line. This can be interpreted as the wavelength dependence of the boundary (the index for longer wavelengths is reduced more by the air boundary than the index for shorter wavelengths). This binary approach therefore suggests that the inclusion of an air boundary imposes a practical limit to the lowest values of dispersion possible. A comparison to Generation 2 shows that Generation 2 is near this practical limit, but with much more easily fabricable cross-sections.


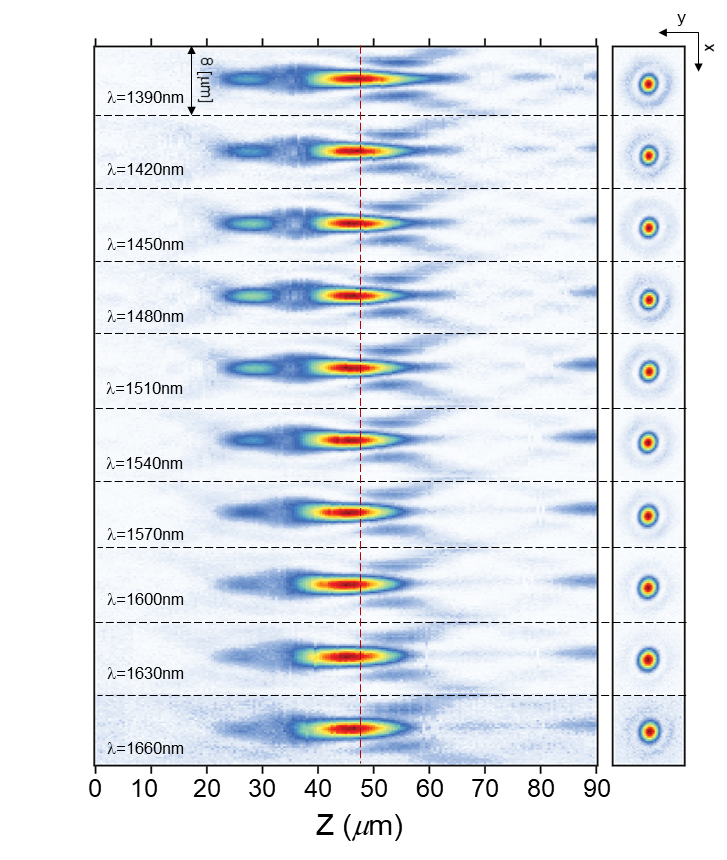
**X. Performance of diverging metalenses**

**Fig. S10 Diverging Lens.** Measured axial (x-z cross-section) and focal plane (x-y cross-section) intensity distributions of a diverging lens with design focal length 50 µm and diameter 100 µm (*NA* ~ 0.7). The measured focal spot is a virtual spot behind the metasurface, where the wavefront originates. Hence, the motorized stage with the objective and the camera is moved towards the metasurface to scan the virtual 3D far-field intensity distribution. The operational bandwidth (~ 270 nm) of the lens is reduced to achieve large *NA*.

**Table S1.** Parameters of fabricated metalenses.

| Metalens | Diameter, *D* (µm) | Focal length, *f* (µm) | Numerical Aperture, *NA* | Library Generation | Operating Bandwidth (nm) |
| --- | --- | --- | --- | --- | --- |
| M1A | 100 | 200 | 0.24 | 1A | 1,300 – 1,650 |
| M1B | 100 | 200 | 0.24 | 1B | 1,200 – 1,650 |
| M2 | 200 | 800 | 0.13 | 1B | 1,200 – 1,650 |
| M3 | 100 | 30 | 0.88 | 2 | 1,200 – 1,400 |
